# Cancer mortality-to-incidence ratio among Iraqi citizens: Nine-year National Estimates (2012-2020) and its relation to population growth rate and health expenditure

**DOI:** 10.5339/qmj.2023.38

**Published:** 2024-01-15

**Authors:** Jasim N Al-Asadi, Jasim M Salman

**Affiliations:** College of Medicine, University of Basrah Email: jnk5511@yahoo.com ORCID iD: 0000-0003-1507-9738

**Keywords:** Incidence, mortality, mortality-to-incidence ratio, cancer, survival, Iraq

## Abstract

Background: Cancer continues to be a significant worldwide health concern with substantial mortality. The cancer mortality-to-incidence ratio (MIR), a proxy measure of observed five-year survival, can serve as a valuable indicator of cancer management outcomes and healthcare disparities among countries. This study aims to determine the MIR trend for all cancers combined among Iraqi citizens during 2012-2020 for health expenditure percentages out of the gross domestic product (e/GDP (%)) and population growth rate.

Methods: The study used the Iraqi Cancer Registry annual reports for cancer data and World Bank data for health expenditure and population growth. Simple linear regression analysis examined the relationship between health expenditure, growth rate, and MIR, while joinpoint regression analysis examined the trend over time. The Ethics Committee of the College of Medicine at the University of Basrah approved the study.

Results: An increasing trend in crude incidence rates for all cancer types combined was seen with a decrease in mortality rates from 2012 to 2020 in both sexes. A non-statistically significant reduction in MIR was found with an average annual percent change (AAPC) of -3.1% (P = 0.100). The decrease in MIR was higher among females than males, with a statistically significant difference (P = 0.003). High health expenditure presented as e/GDP (%) was associated with a favorable cancer survival rate, but this was not statistically significant (R^2^ = 0.263, P = 0.158). In contrast, a low growth rate was significantly associated with cancer patients’ survival (R^2^ = 0.505, P = 0.032).

Conclusions: As indicated by the MIR and the MIR complement (1-MIR), the proxy five-year survival rate is improving in Iraq with time. Although not statistically significant, high health expenditure favorably affected overall cancer survival. A low growth rate, on the other hand, significantly improves cancer patients’ survival.

## Introduction

Globally, there has been a rapid increase in both the incidence and mortality rates of cancer patients attributed to various risk factors, including population growth, aging, and socioeconomic development. Studies reveal that individuals residing in industrialized nations have a two to three times higher chance of developing cancer compared to those in underdeveloped countries. This is primarily due to differences in life expectancy, educational achievement, wealth, early detection and treatment of cancer, and improved registration.^1,2^ However, around 70% of cancer-related deaths occur in low- and middle-income countries. Numerous studies and data from cancer registry reports indicated that Iraqi people are at an increased risk for developing cancer.^3,4^

The incidence rate (IR) of cancer in Iraq has increased from 38.91 per 100,000 people in 1994 to 78.93 per 100,000 people in 2020.^5^ The actual cause of this apparent increase is uncertain, at least for a few cancer types. However, the implementation of early detection programs for specific cancers or improved diagnosis and reporting, population aging, lifestyle changes, environmental damage caused by wars, and economic sanctions are probable factors.^3,6^

Health policymakers must develop programs utilizing epidemiological indices to calculate the disease burden in the community to control, prevent, and treat cancer.^7^ The incidence rate, death rate, and population-based cancer survival are three indices that make up a crucial instrument for estimating the burden of cancer. Moreover, variations in these indicators over time can reflect healthcare quality.^8^

The mortality-to-incidence ratio (MIR) is an index that assesses the impact of cancer on the community and illustrates how well the healthcare system performs concerning patient care and cancer outcomes.^9^ The International Agency for Research on Cancer Registration (IARCR) manual proposed that if cancer registries could not estimate survival directly through comprehensive follow-up of all patients with cancer who had been registered to determine their vital status, the MIR could be used as an alternative indicator of survival.^10^

The lack of active monitoring through population-based cancer registries, particularly in developing countries, hinders efforts to build reliable five-year cancer survival estimates. As a result, several studies examined the validity of MIR or MIR complement [1-MIR] as a valuable predictor of cancer survival. In their research, Sunkara and Hebert described the MIR as a helpful indicator for cancer screening and care in colorectal cancer patients.^11^ Similarly, Stenning-Persivale et al. reported that the 1-MIR is an appropriate tool for approximating observed five-year survival for the ten types of cancers studied.^12^ Ellis et al., on the other hand, stated that the inherent variability in the sensitivity of the MIR to changes in survival and the level of survival at any time since diagnosis between cancers of different lethality invalidates the 1-MIR as a survival measure.^13^

The likelihood of a patient surviving cancer is significantly increased by earlier detection and more effective treatments.^14^ However, more expensive healthcare is needed for screening tests and more potent treatments. Moreover, public health organizations may become overburdened and unable to offer adequate care as the population increases.^15^

We assumed that countries with low growth rates and higher total health expenditure out of the gross domestic product (e/GDP) would have favorable cancer MIR, as recent studies on specific cancers have supported this idea.^16-18^

Iraq is categorized as an upper-middle-income country. Iraq’s economy has suffered decades of political unrest and fluctuating oil prices, resulting in exceptional challenges and damage to the health system. However, over the last decade, Iraq has witnessed some improvements in its health outcomes despite the conflicts.^19^ According to World Bank Data, the per capita health expenditure increased from 173.19 USD in 2012 to 202.31 USD in 2020),^20^ and the e/GDP grew from 2.69% in 2012 to 5.08% in 2020.^21^ Table 1 displays that the annual population growth rate (%) decreased from 4.5 in 2012 to 2.4 in 2020.

To the best of the authors’ knowledge, no previous study in Iraq has used data from population-based cancer registries to estimate the national survival rate of all cancers combined. Therefore, this study was conducted to determine a nine-year time trend (2012-2020) of the MIR for all cancer patients combined in Iraq as an alternative survival measure and the impact of health expenditure presented as e/GDP (%) and population growth rate on it.

## Materials and Methods

### Study Design

This is a retrospective, registry-based study that includes data on cancer cases and deaths reported during the period 2012-2020.

### Data Sources and Collection

The data used in this study was obtained through a review of the official Iraqi Cancer Registry (ICR) annual reports, which are publicly available at (https://moh.gov.iq/?page=35).

The primary anatomical sites of all cancer types were identified and coded according to the International Classification of Diseases for Oncology, Third Edition (ICD-O-3). The reported data included cancer incidence and mortality rates by sex and type of cancer recorded by the Iraqi Cancer Board, Ministry of Health and Environment, for 2012-2020. They are exclusive to Iraqi nationals and do not apply to expatriates working in Iraq. The data for Iraq’s health expenditure out of GDP (e/GDP (%)) during the studied years was obtained from the World Bank Data.^21^ The data for the Iraqi population growth (annual %) during the studied years was obtained from the World Bank Data.^22^

### Definition of Indicators

***Gross domestic product (GDP):*** It is “an economic indicator that measures the monetary value of the total goods and services produced within the borders of the country during a specific period (typically one year)”.^23^

***Health expenditure as a percentage of the gross domestic product (e/GDP (%))*** is the percentage of total general government expenditure on health.^24^

***Population growth rate*** refers to the ratio between the annual change in the population size and the total population for that year, usually multiplied by 100.^25^

### Ethical Approval

The Ethical Committee of the College of Medicine, University of Basrah, approved the study (Project ID: 030409-007-2023).

### Statistical Analysis

All incidence and death rates were crude rates and expressed per 100,000 persons. The cancer incidence rate for each calendar year of the study refers to the patients diagnosed with cancer in that year, depending on pathology reports. The cancer mortality rates were presented for people certified as having died from cancer in that year.

The MIR for all cancer types combined was calculated by dividing the crude mortality rate by the crude incidence rate for all cancer types for each year of the study period and comparing them to the annual population growth rate and e/GDP (%). The 1-MIR was evaluated as a proxy measure for the 5-year relative survival in the same calendar period for all cancer types combined registries.^11^ The median was used to measure the central tendency to obtain an overall assessment of the distribution of the MIR. It was calculated as total and for males and females separately.

The AAPC in MIR was computed to evaluate the magnitude and direction of the trends using the National Cancer Institute’s Joinpoint Regression software program (version 4.9.1.0).^26^

A simple linear regression analysis was done using the IBM Statistical Package for the Social Sciences (SPSS) for Windows, Version 24.0. (IBM Corp., Armonk, N.Y., USA), taking the MIR as the dependent variable and annual population growth rate and e/GDP (%) as the independent variables. P values <0.05 were considered statistically significant. Scatter plots were made using Microsoft Excel 2019.

## Results

The overall incidence rate of cancers during the 9-year study period was 74.38/100,000 population (64.31 for males and 84.71 for females, with a female-to-male ratio of 1.32:1). It increased significantly from 61.69 in 2012 to 78.93 in 2020, with an AAPC of 4.1% (P = 0.002). 

The overall mortality rate was 24.57/100,000 population (24.74 for males and 21.24 for females). It can be seen in Table 2 that it decreased from 30.05 in 2012 to 26.46 in 2020, with an AAPP of -0.2 (P = 0.960).

**Figure 1.**
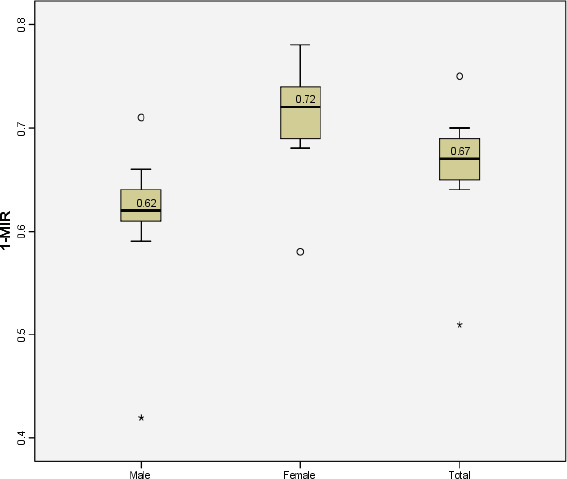
The median of 1-MIR (cancer survival) estimates (Total and by sex)

The overall study period (both sexes) median MIR for all cancers combined was 0.33 (0.38 for males and 0.28 for females). The overall median survival estimates for all cancers combined for both sexes, as reflected by the MIR complement (1-MIR), was 0.67 (67%). It was significantly higher for females than males [0.72 (72%) and 0.62 (62%), respectively, P = 0.003] (Figure 1).

**Figure 2.**
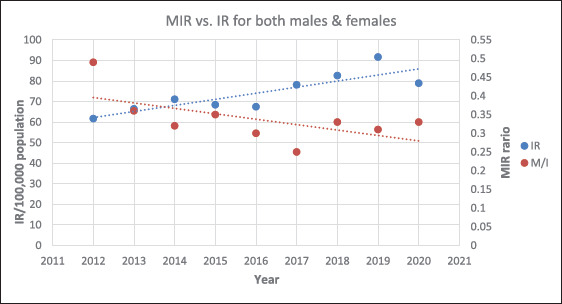
The overall cancer mortality-to-incidence ratio (MIR) vs. incidence rate (IR) per year

No statistically significant decrease was noticed in the MIR over time. It decreased from 0.49 in 2012 to 0.33 in 2020 with an AAPC of -3.1 (P = 0.400). In contrast, the incidence rate increased with time (Figure 2).

**Figure 3.**
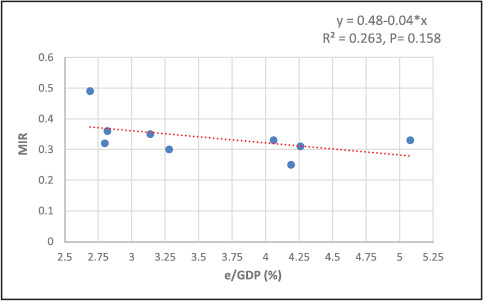
Association of survival with health expenditure/GDP (%) MIR = mortality/incidence ratio, e/GDP = health expenditure as a percentage of the gross domestic product.

The MIR estimates were negatively but not significantly associated with e/GDP (R^2^ = 0.263, P = 0.158). This means that the more resources are allocated to health, the more patients diagnosed with cancer will survive. The regression is Y = 0.48-0.04*x. For a 1-unit increase in e/GDP, there is a 0.04-unit decrement in MIR (Figure 3).

**Figure 4.**
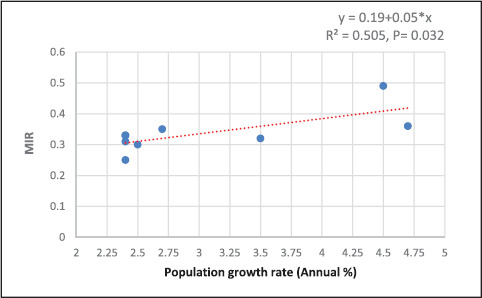
The association of mortality-to-incidence ratio (MIR) with population growth rate (Annual %)

Figure 4 demonstrates a statistically significant positive association between the annual population growth rate and a higher overall cancer MIR with an R^2^ value of 0.505, P = 0.032 (meaning that the annual growth rate explained 50.5% of the total variability in the MIR). The regression formula is Y = 0.19+0.05*x. For every 1 unit increase in growth rate, there was a 0.05 unit increment in MIR.

## Discussion

Understanding population survival is critical both for individuals and public health. Given the scarcity of comprehensive population survival studies, estimating survival based on the complement of mortality and incidence ratios is an option.^9^

This study revealed that during the study period (2012–2020), the overall trend of MIR has not significantly decreased, with an AAPC of -3.1%

p = 0.400, indicating an increase in survival for all cancer patients combined. The median of the MIR of all cancers combined in Iraq for that period was 0.33 for both males and females (0.38 for males and 0.28 for females), giving an overall MIR complement (1-MIR) or a proxy 5-year survival rate of 0.67 or 67% [0.62 (62%) for males and 0.72 (72%) for females]. It is better than that reported for Brazil from 2002 to 2014, which was 52% for males and 56% for females.^27^ This difference could be partly explained by the fact that our study looked at a different time frame than the Brazilian study, or it might result from an improvement in the quality of treatment. Our results are comparable to that in Australia. Based on data sourced from the Australian Institute of Health and Welfare Cancer (2020), the overall MIR for all cancers combined for the period 2012–2019 was 0.34 for both males and females, 0.35 for males, and 0.33 for females, giving a survival rate of 66%, 65%, and 67%, respectively.^28^

The level of completeness of Iraqi death certificates may preclude a meaningful comparison. Nevertheless, the reliability of death registration would be questioned when a condition such as a constant mortality rate was reported.^29^ Additionally, the survivorship of diagnosed cancer cases that have been officially registered in the ICR is estimated through cancer patient follow-up and routine reviewing of the relevant death records.^30^ Even so, it is still possible that the actual number of cancer deaths was underestimated.

Our study found that the survival was statistically higher for female than male cancer patients (the medians were respectively 72% versus 62%, p = 0.003). This result is in agreement with what was reported by Zhua Y et al., who indicated that male cancer patients have higher mortality rates and shorter survival times than female patients.^31^ The observed differences have complex causes, but they can be attributed to behavioral factors such as smoking and alcohol consumption, delayed diagnosis, sex chromosomes, and sex-biased molecular changes.^32^ A negative but not statistically significant linear relationship was found between the e/GDP and the MIR (R^2^ = 0.263, P = 0.158). A similar result was reported by Lee et al., who found no statistically significant association between pancreatic cancer MIR and e/GDP.^33^ However, Ades et al. observed that the more budget spent on health, the fewer the deaths of cancers, and a statistically significant correlation was found between MIRs of all cancers and % e/GDP (r = -0.4726, P = 0.013).^24^ Similarly, Batouli et al. found that cancer MIR in high-income countries (0.47) was significantly lower than that of middle/low-income countries (0.64), with a p<0.001.^34^ In high-income countries, the total health expenditure showed a statistically significant inverse relationship with the overall cancer MIR (P <0.001). Better or more frequent screening programs in countries with higher e/GDP lead to increased cancer diagnosis, early detection, and treatment, thus raising the reported incidence and lowering mortality.^33^

The annual population growth rate and MIR were found to have a statistically significant positive relationship (R^2^ = 0.505, P = 0.032). The relationship between population growth and MIR is complex and multifactorial. Longer life expectancy and lower birth rates, on the other hand, are associated with an aging population, which has an impact on the extent of cancer incidence.^35^ While mortality from noncommunicable diseases increases with age, such as cardiovascular disease, the age-related increase in cancer mortality appears to be slowing.^36^ Hashim et al. reported that the mortality rate for most cancers stabilized or decreased after the age of 85, particularly for non-hormonal cancers. Whether this represents an organic leveling of mortality rates or a reduction in the validity of most cancers’ registration at the various oldest old is debatable.^37^ Similarly, Caroli et al. observed that between 1970 and 2015, the age-standardized mortality rates for all cancers combined showed a heterogeneous but widespread decline in their study of the mortality time trends of 17 cancer types in 11 countries.^38^

It seems that general senility, which restricts cell proliferative potential and the angiogenesis necessary for tumor growth, affects the severity of cancer in old age.^36^ The factors that could influence the patterns of cancer mortality among elderly patients include the presence of comorbidities, less intensive screening, reduced aggressive treatment, disease misclassification, and alterations in the underlying risk factors like hormones.^37^

There are a few limitations to consider with this study. Firstly, there may be some incompleteness in death registries, which cannot be completely ruled out. Secondly, some inaccuracies about the cause of death may occur, particularly among the elderly. Most cancer patients do not die as a result of their disease, and for those who do die, the duration of survival varies widely.^39^

Despite these limitations, this is the first study that establishes the MIR of cancers and its 9-year trend in Iraq, and it offers a distinctive viewpoint on the relationship between MIR of cancers and e/GDP and annual population growth rate. MIR is a simple and quick indicator that can provide important information relevant to the local impact of cancer and be applied as a relative marker of cancer care and the performance of a country’s overall health system,^11^ even though its exact role is still debatable because it would never replace the importance of survival data from cohort surveys.^13^

## Conclusion

Following the findings of previous studies in some countries,^40,41^ the results of this study showed that the incidence of cancer in Iraq during 2012-2020 increased while there was a decrease in mortality rates. As indicated by the MIR and the MIR complement (1-MIR), the proxy five-year survival rate is improving in Iraq with time. Females showed significantly better cancer outcomes than males.

High health expenditure as a percentage of GDP favorably affected overall cancer survival, though this relationship was not statistically significant. While a low growth rate significantly increases cancer patient survival. The findings of this study could help policymakers evaluate current laws and develop effective cancer intervention strategies. Future cohort survival analysis research is required to assess the reliability of MIR in predicting the five-year survival of cancer patients in Iraq.

## Source of Funding

None.

## Conflict of Interest

None of the authors declares a conflict of interest.

## Figures and Tables

**Table 1.** Certain Iraqi economy and health expenditure indicators during 2012-2020

Year	GDP (Billion USD)	GDP per capita (USD)	Per capita health expenditure (USD)	e/GDP (%)	Annual population growth rate (%)
2012	218.00	6,437.5	173.19	2.69	4.5
2013	234.64	6,612.9	186.44	2.82	4.7
2014	228.42	6,219.0	178.54	2.87	3.5
2015	166.77	4,416.9	147.76	3.14	2.7
2016	166.60	4,305.2	142.00	3.28	2.5
2017	187.22	4,725.5	203.17	4.19	2.4
2018	227.37	5,621.2	226.69	4.06	2.4
2019	233.64	5,621.2	239.58	4.26	2.4
2020	180.92	4,251.3	202.31	5.08	2.4

GDP = Gross Domestic Product, e/GDP = Health expenditure out of Gross Domestic Product (quoted from the World Bank Data).

**Table 2.** Overall IR, MR, MIR, 1-MIR for all cancers combined in Iraq during 2012-2020 and by sex

Year	Males							Females						Total					
	Pop.	No. cases	IR	MR	MIR	1-MIR	Pop.	No. cases	IR	MR	MIR	1-MIR	Pop.	No. cases	IR	MR	MIR	1-MIR
2012	17,419,724	9,268	61.69	30.69	0.58	0.42	16,787,524	11,833	53.20	29.38	0.42	0.58	34,207,248	21,101	61.69	30.05	0.49	0.51
2013	17,864,258	10,568	59.16	24.08	0.41	0.59	17,231,514	12,740	73.93	23.45	0.32	0.68	35,095,772	23,308	66.41	23.77	0.36	0.64
2014	18,319,008	11,411	62.29	23.21	0.37	0.63	17,685,544	14,187	80.22	22.38	0.28	0.72	36,004,552	25,598	71.10	22.81	0.32	0.68
2015	18,659,573	11,205	60.05	23.98	0.39	0.61	18,274,141	14,064	76.96	23.80	0.31	0.69	36,933,714	25,269	68.42	23.89	0.35	0.65
2016	19,139,364	11,194	58.49	19.66	0.34	0.66	18,744,179	14,362	76.62	20.30	0.26	0.74	37,883,543	25,556	67.46	19.98	0.30	0.70
2017	18,763,758	12,502	66.63	19.01	0.29	0.71	18,375,761	16,521	89.91	19.47	0.22	0.78	37,139,519	29,023	78.14	19.24	0.25	0.75
2018	19,261,253	13,612	70.67	27.14	0.38	0.62	18,862,929	17,890	94.84	26.85	0.28	0.72	38,124,182	31,502	82.62	26.99	0.33	0.67
2019	19,768,324	15,447	78.14	28.45	0.36	0.64	19,359,565	20,417	105.46	27.55	0.26	0.74	39,127,889	35,864	91.66	28.00	0.31	0.69
2020	20,366,180	13,841	67.96	26.49	0.39	0.61	19,784,020	17,851	90.23	26.42	0.29	0.71	40,150,200	31,692	78.93	26.46	0.33	0.67

IR = incidence rate, MR = mortality rate, MIR = mortality incidence ratio.
